# Beyond the Diagnostic Checklist: A Large‐Scale Analysis of Under‐Recognized Weight Loss Behaviors in Individuals With Eating Disorders

**DOI:** 10.1002/eat.24477

**Published:** 2025-06-17

**Authors:** Saakshi Kakar, Una Foye, Helena L. Davies, Elisavet Palaiologou, Chelsea M. Malouf, Laura Meldrum, Iona Smith, Gursharan Kalsi, Karina L. Allen, Gerome Breen, Moritz Herle, Christopher Hübel

**Affiliations:** ^1^ Social, Genetic & Developmental Psychiatry Centre Institute of Psychiatry, Psychology & Neuroscience, King's College London London UK; ^2^ UK National Institute for Health and Care Research (NIHR) Biomedical Research Centre for Mental Health, South London and Maudsley Hospital London UK; ^3^ Mental Health Nursing, Health Service and Population Research Department Institute of Psychiatry, Psychology & Neuroscience, King's College London London UK; ^4^ Center for Eating and Feeding Disorders Research, Mental Health Center Ballerup Copenhagen University Hospital—Mental Health Services CPH Copenhagen Denmark; ^5^ Institute of Biological Psychiatry, Mental Health Center Sct. Hans, Mental Health Services Copenhagen Roskilde Denmark; ^6^ Eating Disorders Outpatients Service Maudsley Hospital, South London and Maudsley NHS Foundation Trust London UK; ^7^ Department of Psychological Medicine Institute of Psychiatry, Psychology and Neuroscience, King's College London London UK; ^8^ National Centre for Register‐Based Research, Aarhus Business and Social Sciences Aarhus University Aarhus Denmark; ^9^ Clinic for Child and Adolescent Psychiatry, Psychotherapy and Psychosomatics German Red Cross Hospitals Westend Berlin Germany

**Keywords:** anorexia nervosa, binge‐eating disorder, bulimia nervosa, diagnosis, online study, qualitative analysis, text mining, weight loss behavior

## Abstract

**Objective:**

This study aimed to explore the diverse range of weight loss behaviors that extend beyond traditional diagnostic criteria, highlighting the variability in symptom presentation.

**Method:**

We text mined free‐text responses from 1675 participants with anorexia nervosa, bulimia nervosa, or binge‐eating disorder in the Genetic Links to Anxiety and Depression (GLAD) Study and the Eating Disorders Genetics Initiative UK (EDGI UK). In secondary analyses, we investigated differences by eating disorder and gender.

**Results:**

Frequently endorsed behaviors included structured diets (619 endorsements) and calorie counting (422 endorsements), but also less commonly considered behaviors like compression garments (147 endorsements) and self‐harm (88 endorsements). We identified four overarching themes: restriction‐based approaches, medical intervention, body manipulation, and food avoidance. The most frequently reported weight loss behaviors and resultant themes did not differ among eating disorders or genders, closely resembling those in the broader sample. Notably, 81 participants with binge‐eating disorder, which typically lacks the endorsement of recurrent compensatory behaviors, reported weight loss and compensatory behaviors.

**Discussion:**

Our findings identify a crucial gap in current diagnostic assessments, which may hamper recognition and lead to underdiagnosis of eating disorders. By incorporating our insights into an inclusive assessment process that expects and accommodates novel behaviors, clinicians could capture a broader spectrum of behaviors, thus improving diagnostic accuracy. However, our sample homogeneity implies the need for more diverse samples. Our study contributes essential insights for enhancing diagnostic criteria.

**Summary**

- Our study revealed under‐recognized weight loss behaviors in people with eating disorders that current diagnostic tools miss, potentially leading to underdiagnoses.
- By identifying these behaviors and taking a broader diagnostic approach, our research can help clinicians better understand eating disorders by improving diagnostic accuracy and opening up new avenues for personalized care.

## 1 Introduction

Weight loss behaviors are used to try to control body weight or shape (Loth et al. 2014) and constitute core symptoms of many eating disorders. The Diagnostic and Statistical Manual of Mental Disorders, fifth edition (DSM‐5) lists six inappropriate weight loss behaviors for bulimia nervosa: non‐purging behaviors: fasting, excessive exercise, diet pill use; and purging behaviors: self‐induced vomiting, laxative use, and diuretic use (Abebe et al. 2012; American Psychiatric Association 2013). These behaviors may also be present in anorexia nervosa and other specified feeding or eating disorders (OSFED). While purging behaviors are often undertaken to reduce caloric intake, they are largely ineffective, particularly in the case of laxatives and diuretics, whose impact on calorie absorption is minimal (Juarascio et al. 2018). Weight loss behaviors are associated with physical complications, such as cardiomyopathy and rectal prolapse, and psychiatric disorders, including major depressive disorder, generalized anxiety disorder, and suicidality (Campbell and Peebles 2014; Spindler and Milos 2007; Westmoreland et al. 2016).

Eating disorder assessment tools vary in the scope of evaluated behaviors. The ED100K.V3 (Bulik et al. 2021) includes DSM‐5 listed behaviors for bulimia nervosa. Conversely, the Eating Disorder Examination Questionnaire (EDE‐Q 6.0; Fairburn and Beglin 1994) and the Eating Pathology Symptoms Inventory (EPSI; Forbush et al. 2013), two widely recognized assessment tools used in both research and clinic, assess a broader range. The EDE‐Q captures dietary restriction tied to weight and shape concerns, while the EPSI evaluates the exclusion of “unhealthy” and high‐calorie foods from one's diet, alongside the use of muscle‐building supplements, protein supplements, and diet teas. Current assessment tools are limited by their range of assessed weight loss behaviors and their underlying motivations, such as the intent to lose weight.

Semi‐structured interviews are more detailed assessment tools used by researchers and clinicians to diagnose eating disorders. The Eating Disorder Examination (EDE) examines food restrictions (i.e., limitations on quantity, type, and specific food rules) as well as the use of diuretics, laxatives, self‐induced vomiting, and excessive exercise (Fairburn et al. 2008). It also assesses other noteworthy dysfunctional weight‐control behaviors (e.g., spitting, insulin underuse, thyroid medication misuse), requesting details on their frequency (number of days) and nature. The Eating Disorder Assessment for DSM‐5 (EDA‐5) is a web‐based interview that evaluates behaviors associated with bulimia nervosa as outlined in the DSM‐5, as well as other behaviors such as dietary restriction, stimulant use, and spitting food. It also prompts respondents to describe any additional actions they may engage in to manage their weight (Sysko et al. 2015). While these interviews are more robust, they are resource‐intensive, requiring an interviewer with appropriate training and 16.5–35.4 min to administer (Sysko et al. 2015).

Non‐purging and purging weight loss behaviors vary across community and clinical populations. While prevalence is higher in individuals with eating disorders, these behaviors are prevalent in the general population in individuals as young as 12 years old (Forman‐Hoffman 2004; Gonsalves et al. 2014; Neumark‐Sztainer et al. 2006; Solmi et al. 2021). In the US, 34% of middle school students reported using weight loss methods (Yeatts et al. 2016).

The DSM‐5 acknowledges additional harmful behaviors that interfere with weight gain, such as dieting, the misuse of medication, and misuse of enemas (American Psychiatric Association 2013). It acknowledges that individuals with anorexia nervosa and bulimia nervosa may misuse medications, such as manipulating insulin dosages or thyroid medication, as a method to control weight. In contrast, individuals with binge‐eating disorder do not regularly engage in compensatory behaviors (such as purging or excessive exercise), although they may begin dieting after the onset of binge‐eating episodes (American Psychiatric Association 2013). However, these additional associated behaviors are not systematically assessed through eating disorder assessment tools in research.

### 1.1 Weight Loss Behaviors Not Captured by the DSM‐5

Few studies have examined weight loss behaviors in individuals with eating disorders beyond those included in the DSM‐5. For example, individuals with self‐reported eating disorders endorse vaping for appetite and weight control more often than those without eating disorders (Morean and L'Insalata 2017). Chewing and spitting was reported by a quarter of 359 individuals with an eating disorder (Song et al. 2015). Nevertheless, these studies may scratch the surface of the complexity of weight loss behaviors, suggesting a limitation of the behaviors in diagnostic criteria.

Research and clinical practice are limited by diagnostic criteria. Qualitative research enables in‐depth identification of unknown behaviors, uncovering a broader range and providing deeper insights into individuals' experiences and unmet needs. We aimed to identify and categorize the diverse range of weight loss behaviors that extend beyond traditional diagnostic criteria, highlighting the variability in symptom presentation. This work seeks to emphasize the importance of an open‐minded and flexible approach by clinicians when assessing individuals for harmful eating behaviors.

## 2 Methods

### 2.1 Study Design and Sample

Data were collected as part of the Genetic Links to Anxiety and Depression (GLAD; gladstudy.org.uk) Study and Eating Disorders Genetics Initiative UK (EDGI UK; edgiuk.org). Both are ongoing online studies collecting genetic and phenotypic data from UK‐based individuals over 16 years old. The studies form part of the National Institute for Health and Care Research (NIHR) Mental Health BioResource; a data resource comprising recontactable participants. Further details about study processes are described elsewhere (Davies et al. 2019; Monssen et al. 2024). For details of the recruitment process, see Supporting Information Methods.

The GLAD Study investigates the genetic and environmental bases of anxiety and depressive disorders (Davies et al. 2019). Participants live in the United Kingdom and either have a lifetime experience of anxiety and/or depressive disorders (cases) or have never experienced any mental health conditions (controls). Present analyses comprise responses from cases to the optional ED100K.V3 questionnaire, collected on or before September 16, 2022 (Thornton et al. 2018).

EDGI UK launched in February 2020 to recruit 10 000 participants who have ever experienced an eating disorder (Monssen et al. 2024). Participants are residents of England with a lifetime experience of eating disorders. Present analyses consist of all sign‐up questionnaires completed on or before September 16, 2022.

The London—Fulham Research Ethics Committee approved the GLAD Study on August 21, 2018 (REC reference: 18/LO/1218) and EDGI UK on July 29, 2019 (REC reference: 19/LO/1254).

### 2.2 Measures

Demographic characteristics (age, sex assigned at birth, gender, ethnicity, highest level of education, sexuality) were collected through the GLAD Study and EDGI UK sign‐up questionnaires.

Lifetime eating disorder diagnoses were self‐reported in the GLAD Study and EDGI UK via the question, “Have you been diagnosed with one or more of the following mental health problems by a professional, even if you don't have it currently?” with eating disorders as response options as listed in the DSM‐5 (American Psychiatric Association 2013; Davis et al. 2020).

Eating disorder diagnoses were additionally determined using diagnostic algorithms, based on responses to the ED100K.V3, using DSM‐5 criteria (American Psychiatric Association 2013; Bulik et al. 2021; github.com/tnggroup/EDGI_protocol). The ED100K.V3 is a self‐report questionnaire that assesses lifetime eating disorder symptoms (Bulik et al. 2021), based on the Structured Clinical Interview for DSM‐5, Eating Disorders. The ED100K‐V1 is clinically validated for criteria B and C for anorexia nervosa and for binge eating (Thornton et al. 2018). While an optional questionnaire in the GLAD Study, the ED100K.V3 is the basis of the sign‐up questionnaire in EDGI UK. A hierarchical categorization (anorexia nervosa restricting > anorexia nervosa binge‐eating/purging > anorexia nervosa unknown subtype > bulimia nervosa > binge‐eating disorder) was applied to ensure no overlapping cases among the algorithm‐defined eating disorders. This hierarchy is common practice in epidemiological studies (Schaumberg et al. 2019; Udo and Grilo 2018). For inclusion, participants must have answered the ED100K.V3 (Bulik et al. 2021) and have either a self‐reported or an algorithm‐derived eating disorder: anorexia nervosa, bulimia nervosa, or binge‐eating disorder.

In the ED100K.V3, the free‐text question: “Have you ever used any other methods not listed in the previous question to control your body shape or weight” assessed weight loss behaviors and compensatory behaviors, used to offset binge eating, were assessed via: “Please state any other methods used to compensate for episodes of binge eating or overeating.” (Bulik et al. 2021). Some participants provided details about their weight loss behaviors, which have been paraphrased to protect participant confidentiality and are highlighted in italics in the results section.

### 2.3 Data Analyses

**FIGURE 1:**
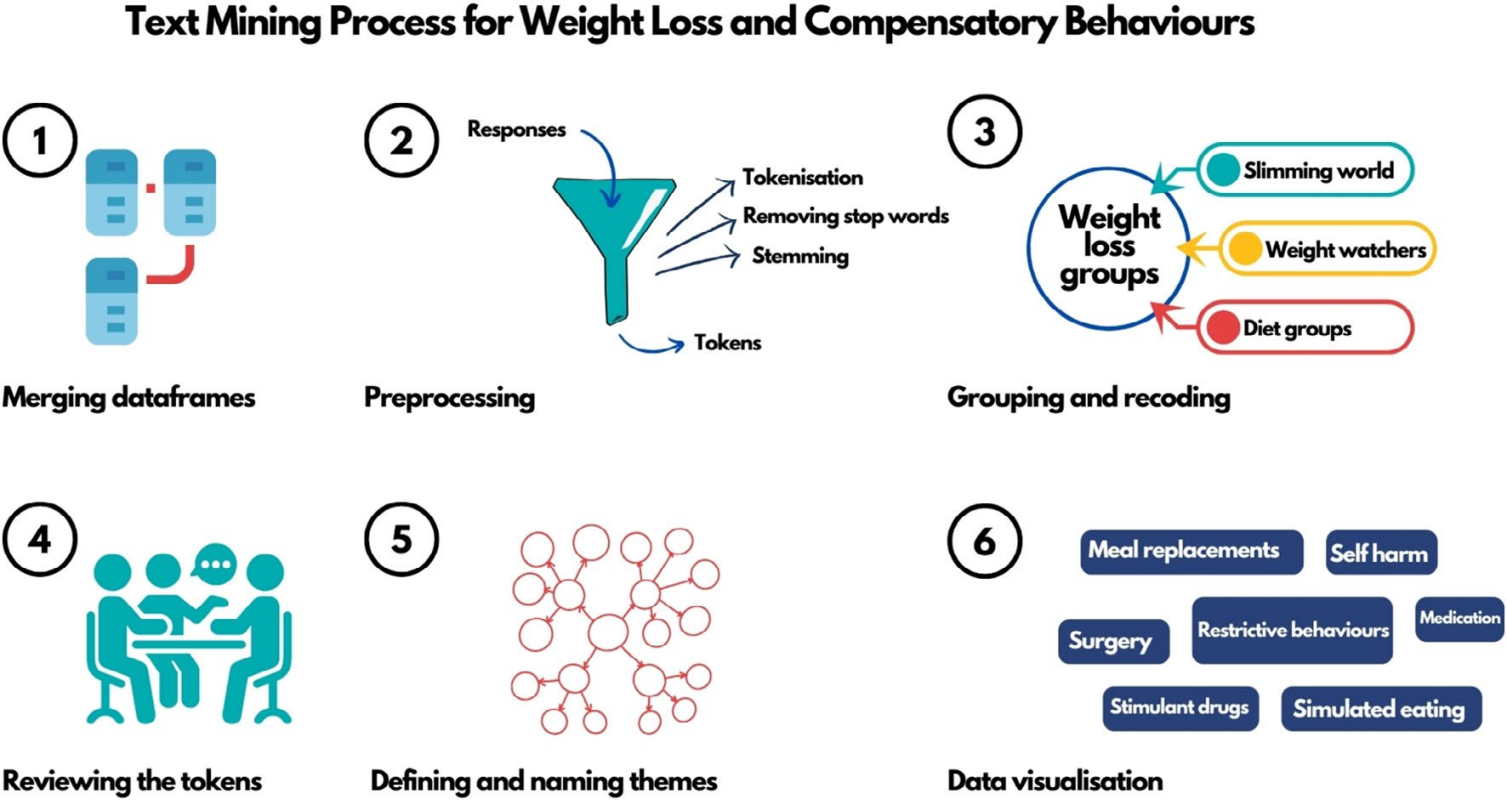
Analysis flowchart of text mining on free‐text responses to weight loss and compensatory behaviors questions from participants of the GLAD Study and EDGI UK.

Prior to analyses, we merged data from the GLAD Study and EDGI UK ED100K.V3. We then cleaned the dataset by excluding participants with missing information, those without self‐reported or algorithm‐derived anorexia nervosa, bulimia nervosa, or binge‐eating disorder, and individuals who did not respond to the free‐text questions regarding weight loss or compensatory behaviors. Descriptive statistics were subsequently reported (Table 1). Analyses were conducted using the R package *tidytext* (Silge and Robinson 2016). We text mined (i.e., extracted patterns from text) to analyze free‐text responses (Figure 1; Jo 2019). We used a thematic analysis approach (Braun and Clarke 2006). We made interpretation and coding decisions rather than relying on automated processes. The qualitative nature of our work ensures that the identified themes reflect the context and meaning expressed by the participants.

**TABLE 1:** Demographic characteristics of the GLAD and EDGI UK sample that answered the ED100K.V3 (*n* = 1675).

Characteristic	*n*	%
Sex		
Female	1588	94.8%
Male	87	5.2%
Gender		
Woman	1510	90.2%
Man	99	5.9%
Non‐binary	42	2.5%
Self‐defined	13	0.8%
Don't know/Prefer not to say	11	0.7%
Eating disorder diagnosis		
Anorexia nervosa		
Restricting	231	13.8%
Binge‐eating/purging	452	27.0%
Unknown subtype	8	0.5%
Bulimia nervosa	903	53.9%
Binge‐eating disorder	81	4.8%
Ethnicity		
White	1568	93.6%
Mixed	61	3.6%
Asian or Asian British	17	1.0%
Black, Black British, or Arab	8	0.5%
Don't know/other	21	1.1%
Highest level of education		
University	825	49.3%
A‐levels/AS‐levels or equivalent	487	29.1%
NVQ/HND/HNC or equivalent	103	6.2%
GCSEs/CSEs/O‐levels or equivalent	200	11.9%
None of the above	42	2.5%
Seen but not answered	18	1.1%
Sexuality		
Heterosexual	1024	61.1%
Homosexual	96	5.7%
Bisexual	389	23.2%
Asexual	40	2.4%
Self‐defined	48	2.9%
Other	46	2.8%
Prefer not to answer	32	1.9%

*Note*: Eating disorder diagnoses are either algorithm‐derived or self‐reported. The unknown subtype of anorexia nervosa results from the self‐report questionnaire not differentiating between subtypes in the GLAD study. Due to low numbers, we created a merged category “Black, Black British, or Arab” to protect privacy.

**1. Merging data frames:** We merged data from the GLAD Study and EDGI UK, creating separate data frames for responses discussing weight loss behaviors and compensatory behaviors (i.e., following binge eating).

**2. Preprocessing**: We performed preprocessing operations to prepare the data frames for analysis.

**2.1. Tokenisation**: Splitting the text into meaningful units (in this case, sentences).

**2.2. Stop word removal**: Eliminating words like “a” and “the” that do not have semantic value for the analysis.

**2.3 Stemming**: Reducing words to their root form.

**3. Categorizing and recoding**: We categorized and recoded sentence tokens referencing the same or similar weight loss and compensatory behaviors into codes. For instance, responses like “slimming world” and “weight watchers” were combined and recoded as “weight loss groups”.

**3.1. Handling multiple behaviors in a token**: When a participant endorsed more than one behavior in a token, the responses were recoded with a full stop between meaningful chunks to allow separate tokenisation and individual analysis. For example, “Smoking and self‐harm” was recoded to “Smoking. Self‐harm.”, resulting in more than one token per participant. We assessed the overlap of behaviors through a co‐occurrence network graph.

**3.2. Combining multi‐sentence behaviors**: When participants described one behavior across multiple sentence tokens, the responses were recoded into a single token to avoid double‐counting. For example, “Chew gum instead of food. Excessively” was recoded to “Chew gum instead of food”.

**3.3. Calculating endorsement of behavior tokens:** To determine the percentage of endorsement for each weight loss and compensatory behavior, we calculated the proportion of tokens endorsing a specific behavior relative to the total number of response tokens per question.

**4. Reviewing the tokens:** The initial coding was performed by the first author, followed by a second round by EP and HLD using Excel. Any discrepancies were resolved through team discussions.

**5. Defining and naming themes:** We organized the tokens into potential themes and categorized related codes together to form cohesive and meaningful categories that accurately represent the weight loss and compensatory behaviors reported by participants. While some behaviors may fit into multiple themes, we categorized them based on their most prominent characteristics—such as their primary function or intended outcome—to maintain clarity and analytical focus.

**6. Data visualization:** We then created graphics to visually represent the most endorsed behavior themes.

In secondary analyses, the text mining process outlined above was repeated to examine differences by eating disorder and gender.

1. **Data Segmentation**: The dataset was filtered to isolate responses from participants with anorexia nervosa, bulimia nervosa, and binge‐eating disorder and separately by gender. The subsets were then subjected to the same preprocessing steps as the overall dataset.
2. **Behavioral Categorization**: The same categorization and recoding strategies were applied to categorize behaviors within these subgroups, ensuring consistency with the initial analysis.
3. **Interpretation of Results**: The results of this secondary analysis were compared to the overall findings, providing insights into how individuals with different eating disorder diagnoses and men may exhibit distinct behaviors within the context of the study.

## 3 Results

The total sample consisted of 1675 participants, 94.8% female, from the GLAD Study and EDGI UK (Table 1) aged on average 31.7 years (SD = 13.1; range = 16–77). In the sample, 27% had a diagnosis of anorexia nervosa binge‐eating/purging and 13.8% had a restricting subtype. Additionally, 53.9% of participants had a diagnosis of bulimia nervosa and 4.8% of binge‐eating disorder. In our sample, 53% of participants had both self‐reported and algorithm‐derived eating disorder diagnoses, indicating concordance between the two approaches, with the remaining 47% identified solely through algorithm‐derived diagnoses.

We excluded missing data, including NA and “Seen but not answered” responses: 13 386 participants completed the ED100K but did not respond to the free‐text weight loss behavior question, and an additional 3177 did not complete the free‐text compensatory behavior question. The high number of nonresponses to the free‐text questions may reflect their greater cognitive and time demands compared to Likert scale questions, or alternatively, may indicate that participants had no additional information to provide. After preprocessing, 3492 tokens regarding weight loss behaviors and 815 tokens regarding compensatory behaviors were available for analysis (Table 2).

**TABLE 2:** Frequency of endorsed weight loss behaviors (*n* = 3492 tokens) and compensatory behaviors (*n* = 815 tokens), as well as weight loss behaviors reported by men (*n* = 99 tokens), based on free‐text responses from the ED100K questionnaire in the GLAD Study and EDGI UK.

Behavior tokens	Weight loss behavior endorsement (*n* = 3492)	Compensatory behavior endorsement (*n* = 815 tokens)	Men's endorsement (*n* = 99 tokens)
Structured/popular weight loss diets, e.g., Atkins diet, Cambridge diet	619 (17.7%)	421 (51.7%)	16 (16.1%)
Calorie counting	422 (12.1%)	281 (34.5%)	7 (7.1%)
Restrictive eating, e.g., restrictive food rules	389 (11.1%)	279 (34.2%)	10 (10.1%)
Chewing & spitting	214 (6.1%)	155 (19.0%)	< 5
Illicit drugs, e.g., cocaine, amphetamines	213 (6.1%)	154 (19.0%)	12 (12.1%)
Fluid loading	207 (5.9%)	94 (11.5%)	< 5
Weight loss groups, e.g., slimming world	160 (4.6%)	136 (16.7%)	< 5
Meal replacements, e.g., meal replacement milkshakes	155 (4.4%)	117 (14.4%)	< 5
Compression garments, e.g., corsets	147 (4.2%)	73 (9.0%)	< 5
Bariatric surgery, e.g., gastric bypass, gastric bands	146 (4.2%)	100 (12.3%)	6 (6.1%)
Appetite suppressants, e.g., caffeine, smoking	122 (3.5%)	74 (9.1%)	5
Self‐harm	88 (2.5%)	49 (6.0%)	< 5
Prescription medication, e.g., metformin	81 (2.3%)	59 (7.2%)	6 (6.1%)
Reducing body temperature, e.g., ice baths	68 (1.9%)	23 (2.8%)	< 5
Inducing sweating	47 (1.3%)	33 (4.0%)	0
Consuming foods incompatible with intolerances	47 (1.3%)	21 (2.6%)	< 5
Cognitive methods, e.g., engaging with pro‐anorexia/pro‐bulimia websites, body checking, hypnotherapy, mindfulness, negative imagery	45 (1.3%)	9 (1.1%)	0
Hiding/disposing of food	37 (1.1%)	23 (2.8%)	0
Restricting fluid intake	33 (0.9%)	21 (2.6%)	0
Micro‐exercise, e.g., leg tapping, pacing, fidgeting	31 (0.9%)	0	0
Sleeping to avoid eating	31 (0.9%)	15 (1.8%)	0
Eating non‐food items, e.g., tissue, wallpaper paste, cotton balls	29 (0.8%)	15 (1.8%)	< 5
Excessive alcohol consumption	26 (0.7%)	12 (1.5%)	< 5
Chewing gum	22 (0.6%)	12 (1.5%)	0
Colon cleansing, e.g., colonic irrigation, enemas	18 (0.5%)	14 (1.7%)	< 5
Electrical muscle stimulation	17 (0.5%)	10 (1.2%)	< 5
Weight loss injections, e.g., saxenda	13 (0.4%)	16 (2.0%)	0
Manipulating a nasogastric tube	11 (0.3%)	16 (2.0%)	0
Sleep deprivation	6 (0.2%)	0	0
Overeating to induce vomiting	5 (0.1%)	0	0
Energy drinks	5 (0.1%)	0	0
Steroids	5 (0.1%)	0	0

**FIGURE 2:**
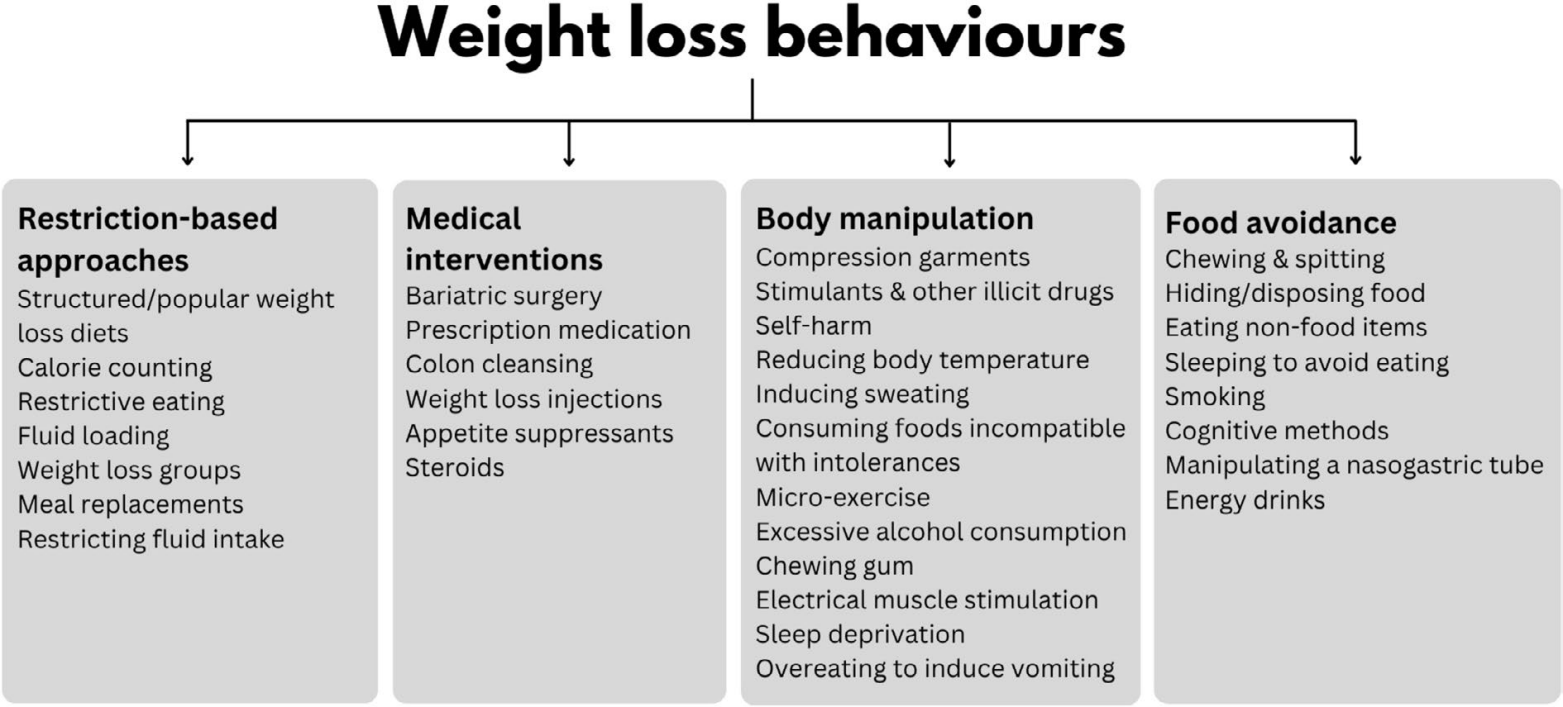
The identified themes from respondents' weight loss behaviors.

**FIGURE 3:**
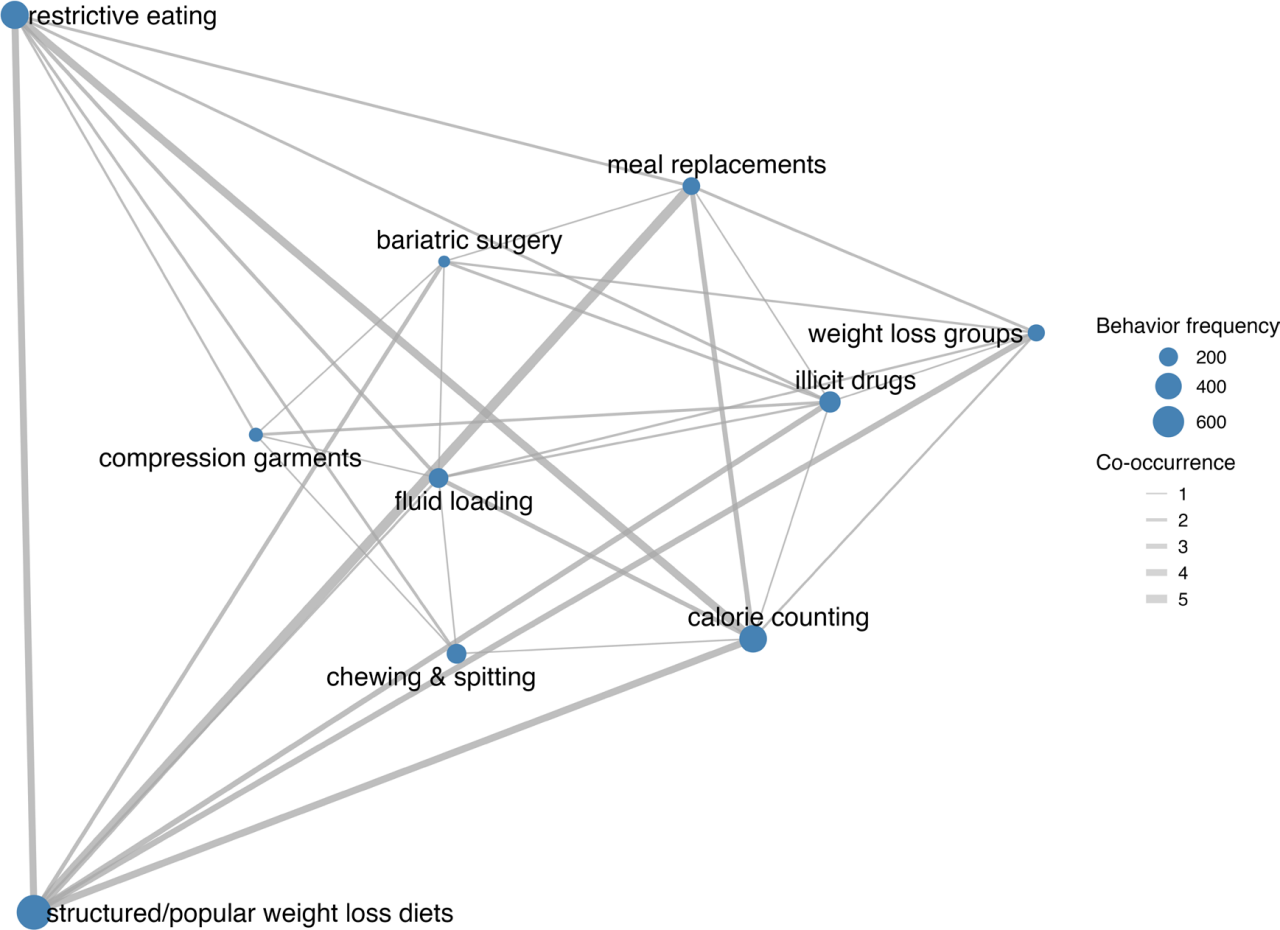
Co‐occurrence network of weight loss behaviors identified in the study. Each node represents a specific behavior, with the node's size corresponding to the frequency of that behavior's occurrence. Edges (lines) between nodes indicate instances where two behaviors co‐occurred, with the thickness of each edge reflecting the strength of the co‐occurrence. This visualization provides insights into how different weight loss behaviors are interrelated, highlighting patterns of behavior combinations among participants.

The weight loss and compensatory behavior tokens were highly similar. Therefore, we categorized both types of tokens into four themes, providing a more structured understanding of the weight control methods: restriction‐based approaches, medical interventions, body manipulation, and food avoidance (Figure 2). These themes highlight the range of strategies used to control weight, from more common dietary methods to less common, more extreme practices not typically captured by standard assessments. Many participants (*n* = 266) reported engaging in a combination of behaviors, resulting in several tokens across themes (Figure 3). Behaviors such as structured/popular weight loss diets, calorie counting, and meal replacements frequently co‐occurred with each other and had less frequent overlap with illicit drugs.

Restriction‐based approaches encompass behaviors focused on controlling food and fluid intake, including diet‐based strategies, such as structured/popular weight loss diets like the Atkins Diet, and participation in weight loss groups like WeightWatchers. Additionally, fluid‐related behaviors, such as consuming excessive amounts of water or carbonated drinks to create a *sense of fullness*, or conversely, restricting fluid intake to avoid feeling bloated and *with the fear it would make them fat*, were noted.

Medical interventions include invasive or pharmaceutical methods including bariatric surgery, such as gastric bypass and gastric band surgery, which restrict the stomach's capacity to hold food. Additionally, participants endorsed using medications like metformin (used to treat type 2 diabetes), methylphenidate (used to treat attention‐deficit/hyperactivity disorder), and levothyroxine (used to treat hypothyroidism) for weight loss rather than their prescribed intent. Participants reported using various substances, including caffeine from coffee or pills, illicit drugs, and smoking, to suppress appetite.

Body manipulation captures physically manipulative methods. Participants described lowering their body temperature through baths, intentionally keeping cold, or inducing sweating by wrapping themselves in cling film or spending extended periods in saunas to *use up calories*. Some individuals engaged in self‐harm, such as cutting or burning fat off their bodies, to directly reduce body weight. Some deliberately consumed foods incompatible with their intolerances, such as eating gluten despite being coeliac, to provoke physical discomfort that discouraged eating, as a form of *punishment* to suppress food intake, or to expend calories. Some participants also engaged in sleep deprivation, viewing it as a strategy to burn calories, while others reported overeating deliberately to induce vomiting as a means of purging. Other methods included engaging in “micro‐exercises,” performing small, repetitive movements throughout the day to burn calories without drawing attention. Excessive alcohol consumption was also mentioned as a tactic to reduce food intake and *make them feel sick*. Finally, respondents wore compression garments, like corsets or waist trainers, to physically restrict the stomach, create a slimmer appearance, and make eating *uncomfortable*.

Food avoidance encompasses behaviors to prevent or reduce food consumption, such as chewing and spitting and, in more extreme cases, consuming non‐food items to increase stomach distension without ingesting calories. Chewing gum was frequently used to create a sensation of fullness, suppress hunger, or provide a distraction from cravings, potentially leveraging the cephalic phase response to simulate the experience of eating without caloric intake.

**FIGURE 4:**
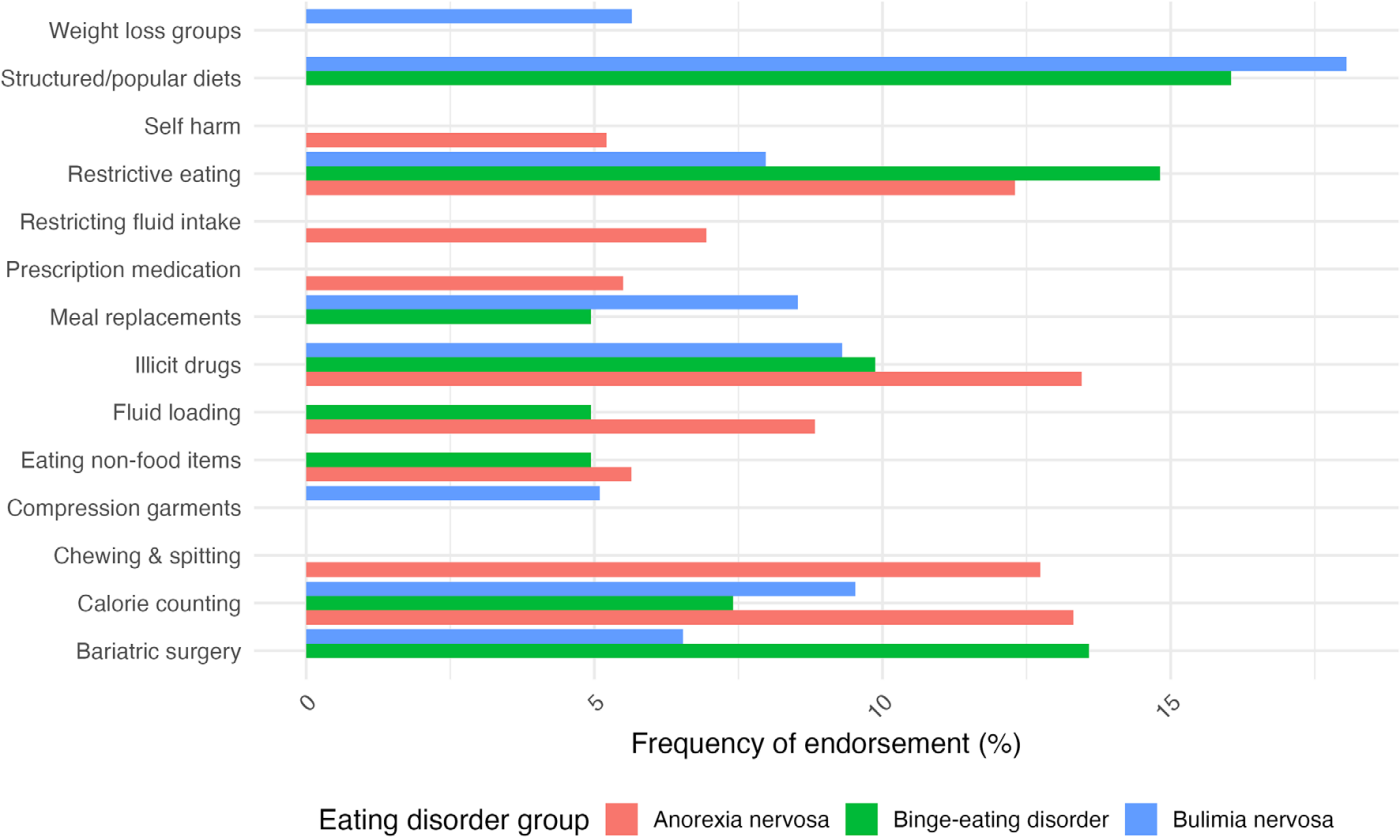
Frequencies of weight loss behaviors by eating disorder: Anorexia nervosa (across subtypes; *n* = 691), bulimia nervosa (*n* = 903), and binge‐eating disorder (*n* = 81). If the bar is not displayed, the weight loss behavior was not endorsed.

In the secondary analysis, we analyzed responses separately for anorexia nervosa (across subtypes; *n* = 691), bulimia nervosa (*n* = 903), and binge‐eating disorder (*n* = 81; Figure 4). Among participants with anorexia nervosa, the three most frequently reported behaviors were calorie counting (13.3%), chewing and spitting (12.7%), and restrictive eating (12.3%). For participants with bulimia nervosa, the top three behaviors were structured/popular weight loss diets (18.1%), calorie counting (9.5%), and meal replacements (8.5%). Among participants with binge‐eating disorder, structured/popular weight loss diets (16.1%), restrictive eating (14.8%), and bariatric surgery (13.6%) were the most frequently reported.

We also focused on men (*n* = 87), of whom 17 (19.5%) identified as transgender men. Of these self‐identified men, 56 (64.4%) reported being homosexual, bisexual, or asexual. After data cleaning, 99 tokens were analyzed. Men endorsed weight loss behaviors closely mirroring the broader sample (Table 2).

## 4 Discussion

In 2025, our study represents the largest systematic analysis of weight loss behaviors that may be overlooked by diagnostic interviews and questionnaires for eating disorders. We identified four themes–restriction‐based approaches, medical interventions, body manipulation, and food avoidance–through text mining over 3000 tokens derived from free‐text responses. Our findings highlight a crucial issue, that individuals with eating disorders often engage in weight loss behaviors that are not included in current diagnostic assessments.

Standard eating disorder assessment tools, such as the EDE‐Q 6.0 and the EPSI, evaluate restriction‐based behaviors like restrictive eating, calorie counting, and detox teas, and medical interventions like the use of steroids (Fairburn and Beglin 1994; Forbush et al. 2013). However, these tools do not account for the full range of behaviors identified in our study, such as the use of non‐food items, extreme food manipulation techniques, or off‐label drug use for weight control. While clinical interviews, such as the EDE interview, offer greater flexibility in assessing target behaviors—including the use of open‐ended questions and prompts to guide interviewer judgment—they are resource‐intensive and may hinder participants in providing sensitive information due to the stigmatized nature of the behaviors (O'Connor et al. 2021). This underscores the need for improved questionnaire‐based assessments that are both scalable and sensitive, particularly for large‐scale studies such as the GLAD Study and EDGI UK.

The gap in the diagnostic framework can lead to missed or incorrect diagnoses, as patients may not disclose certain behaviors unless specifically prompted, often due to the secrecy and shame surrounding eating disorders (Barko and Moorman 2023). Addressing this gap requires shifting focus from predefined behaviors to an inclusive assessment process that expects and accommodates novel behaviors. Engaging individuals with lived experience in the development of assessments can help ensure that these evolving behaviors are recognized and incorporated effectively. Without a comprehensive understanding of all relevant behaviors, clinicians may overlook critical aspects and health risks. For example, consuming non‐food items may result in digestive complications, and off‐label use of prescription medications poses considerable health risks like electrolyte disturbances or heart problems (Hendricks 2017; Kariuki et al. 2016).

A key finding was that 81 participants with binge‐eating disorder engaged in weight loss and compensatory behaviors typically linked to bulimia nervosa. This challenges the clear‐cut boundaries between eating disorders, as DSM‐5 criteria for binge‐eating disorder specify that binge eating must not be accompanied by recurrent inappropriate compensatory behaviors (American Psychiatric Association 2013). The endorsement of medical interventions by those with binge‐eating disorder aligns with research, as bariatric surgery may be pursued as a weight loss method according to national treatment guidelines for obesity (Da Luz et al. 2018). Additionally, individuals with binge‐eating disorder may adopt risky, overlooked weight loss methods, such as eating non‐food items and stimulant drugs, potentially worsening illness severity.

One of our study's strengths lies in its hypothesis‐free qualitative approach encouraging participants to candidly describe their behaviors, allowing for the identification of a broad range of weight loss behaviors not currently covered by diagnostic criteria. The open‐ended format of the two questions in the ED100K.V3 and the questionnaire's completion in private encouraged more candid disclosures from participants. This resulted in rich, authentic data reflecting behaviors people may hesitate to share in clinical settings. Additionally, our study is strengthened by the high number of participants and broad age range.

The study has the following limitations. One limitation of this study is that we did not assess whether the behaviors identified are associated with clinically significant distress, impairment, or risk. While some behaviors (e.g., off‐label medication use) clearly pose health risks, others may be more normative or context dependent. Additionally, the clarity of the free‐text question asking participants to disclose any additional methods used to control their body shape or weight must be considered. For example, several responses described strategies to gain weight, such as marijuana to encourage appetite and protein shakes. Since both the GLAD Study and EDGI UK are ongoing, this question will be refined.

Research suggests that adolescents from minoritised ethnic groups demonstrate similar or higher tendencies to adopt weight loss behaviors compared with white adolescents (Goldschmidt et al. 2008). As our UK sample is mostly white, female, heterosexual, and highly educated, future research should recruit participants from more varied racial, ethnic, gender, and socioeconomic backgrounds. The GLAD Study and EDGI UK have already launched strategic advertising to enhance diversity.

The themes were developed to meaningfully organize the wide range of behaviors reported. However, we acknowledge that the boundaries between these themes are not always clear‐cut. Certain behaviors, such as fluid loading or smoking, could plausibly be categorized under multiple themes—for example, smoking may be considered both a restriction‐based approach and a medical intervention, depending on the individual's intent and context. These overlaps reflect the complexity and nuance of real‐world behaviors and highlight the limitations of strict thematic categorization. We chose to group behaviors based on dominant patterns observed in the data but recognize that individual interpretations may vary. This fluidity reinforces the need for ongoing refinement of behavioral classifications in future research.

Future research should conduct in‐depth interviews to understand motivations, context, and evolution of these weight loss behaviors. Although the broad age range of participants is a strength of the study, future research should conduct age‐specific analyses to explore whether age may have influenced the expression or reporting of these behaviors. It would also be valuable to investigate the prevalence of the identified behaviors across a broader sample, including individuals without diagnosed eating disorders, to better differentiate between weight‐related behaviors that are normative versus those associated with or indicative of eating disorder psychopathology. Additionally, examining the relationship between these behaviors and markers of mental wellness, such as anxiety, depression, and overall health, would help clarify the clinical significance of these behaviors and inform the development of more targeted diagnostic tools and interventions. For example, Aouad et al. (2021) demonstrated that chew and spit behaviors in adolescents were associated with poorer health‐related quality of life and disordered eating, underscoring the importance of recognizing and assessing such behaviors in clinical settings. Future research will also involve collaborating with clinicians to categorize the identified behaviors by health impact to ensure the classification reflects clinical priorities and informs more nuanced assessment and intervention strategies.

## 5 Conclusion

This study uncovered a wide range of weight loss behaviors that are often overlooked by current research and diagnostic frameworks, highlighting the limitations of existing tools. Developing psychometric tools designed to capture these broader behaviors could further enhance detection and facilitate earlier intervention. Future research should aim to diversify the sample and employ detailed interviews to gain a deeper understanding of these behaviors, improving the inclusivity of diagnostic criteria.

## Author Contributions

**Saakshi Kakar:** software, data curation, writing – review and editing, writing – original draft, visualization, formal analysis, validation, resources, investigation, conceptualization. **Una Foye:** conceptualization, investigation, methodology, supervision, visualization, writing – review and editing, writing – original draft. **Helena L. Davies:** data curation, conceptualization, formal analysis, writing – review and editing, investigation, methodology, project administration, software, supervision, validation. **Elisavet Palaiologou:** formal analysis, validation, writing – review and editing. **Chelsea M. Malouf:** data curation, project administration, resources, writing – review and editing. **Laura Meldrum:** data curation, project administration, resources, writing – review and editing. **Iona Smith:** data curation, project administration, resources, writing – review and editing. **Gursharan Kalsi:** data curation, supervision, resources, project administration, writing – review and editing. **Karina L. Allen:** writing – review and editing, supervision, writing – original draft. **Gerome Breen:** writing – review and editing, project administration, conceptualization, funding acquisition, methodology, resources, supervision. **Moritz Herle:** writing – review and editing, writing – original draft, supervision, visualization, conceptualization, investigation, methodology. **Christopher Hübel:** conceptualization, writing – original draft, writing – review and editing, visualization, software, data curation, supervision, validation, investigation, methodology, formal analysis, project administration.

## Ethics Statement

The London—Fulham Research Ethics Committee approved the GLAD Study on August 21, 2018 (REC reference: 18/LO/1218) and EDGI UK on July 29, 2019 (REC reference: 19/LO/1254) following a full review. The NIHR BioResource has been approved as a Research Tissue Bank by the East of England—Cambridge Central Committee (REC reference: 17/EE/0025)

## Conflicts of Interest

Prof. Gerome Breen has received honoraria, research or conference grants and consulting fees from Illumina, Otsuka, and COMPASS Pathfinder Ltd.

## Supporting information


**Data S1.**Supporting Information.

## Data Availability

The data that support the findings of this study are available from NIHR BioResource. Restrictions apply to the availability of these data, which were used under license for this study. Data are available from https://bioresource.nihr.ac.uk/ with the permission of NIHR BioResource.

## References

[eat24477-bib-0001] Abebe, D. S. , L. Lien , L. Torgersen , and T. von Soest . 2012. “Binge Eating, Purging and Non‐Purging Compensatory Behaviours Decrease From Adolescence to Adulthood: A Population‐Based, Longitudinal Study.” BMC Public Health 12: 1–10. 22244266 10.1186/1471-2458-12-32 PMC3298533

[eat24477-bib-0002] American Psychiatric Association . 2013. Diagnostic and Statistical Manual of Mental Disorders. 5th ed. American Psychiatric Association. 10.1176/appi.books.9780890425596.

[eat24477-bib-0003] Aouad, P. , P. Hay , N. Soh , S. Touyz , H. Mannan , and D. Mitchison . 2021. “Chew and Spit (CHSP) in a Large Adolescent Sample: Prevalence, Impact on Health‐Related Quality of Life, and Relation to Other Disordered Eating Features.” Eating Disorders 29, no. 5: 509–522. 10.1080/10640266.2019.1695449. 31770086

[eat24477-bib-0004] Barko, E. B. , and S. M. Moorman . 2023. “Weighing in: Qualitative Explorations of Weight Restoration as Recovery in Anorexia Nervosa.” Journal of Eating Disorders 11, no. 1: 14. 36721222 10.1186/s40337-023-00736-9 PMC9887881

[eat24477-bib-0005] Braun, V. , and V. Clarke . 2006. “Using Thematic Analysis in Psychology.” Qualitative Research in Psychology 3, no. 2: 77–101.

[eat24477-bib-0006] Bulik, C. M. , L. M. Thornton , R. Parker , et al. 2021. “The Eating Disorders Genetics Initiative (EDGI): Study Protocol.” BMC Psychiatry 21: 1–9. 33947359 10.1186/s12888-021-03212-3 PMC8097919

[eat24477-bib-0007] Campbell, K. , and R. Peebles . 2014. “Eating Disorders in Children and Adolescents: State of the Art Review.” Pediatrics 134, no. 3: 582–592. 10.1542/peds.2014-0194. 25157017

[eat24477-bib-0008] Da Luz, F. Q. , P. Hay , S. Touyz , and A. Sainsbury . 2018. “Obesity With Comorbid Eating Disorders: Associated Health Risks and Treatment Approaches.” Nutrients 10, no. 7: 829. 29954056 10.3390/nu10070829 PMC6073367

[eat24477-bib-0009] Davies, M. R. , G. Kalsi , C. Armour , et al. 2019. “The Genetic Links to Anxiety and Depression (GLAD) Study: Online Recruitment Into the Largest Recontactable Study of Depression and Anxiety.” Behaviour Research and Therapy 123: 103503. 31715324 10.1016/j.brat.2019.103503 PMC6891252

[eat24477-bib-0010] Davis, K. A. , J. R. Coleman , M. Adams , et al. 2020. “Mental Health in UK Biobank–Development, Implementation and Results From an Online Questionnaire Completed by 157 366 Participants: A Reanalysis.” BJPsych Open 6, no. 2: e18. 32026800 10.1192/bjo.2019.100 PMC7176892

[eat24477-bib-0012] Fairburn, C. , Z. Cooper , and M. O'Connor . 2008. “Eating Disorder Examination (Edition 16.0D).” In Eating Disorders and Cognitive Behavior Therapy, 265–308. Guilford Press.

[eat24477-bib-0013] Fairburn, C. G. , and S. J. Beglin . 1994. “Assessment of Eating Disorders: Interview or Self‐Report Questionnaire?” International Journal of Eating Disorders 16, no. 4: 363–370. 7866415

[eat24477-bib-0014] Forbush, K. T. , J. E. Wildes , L. O. Pollack , et al. 2013. “Development and Validation of the Eating Pathology Symptoms Inventory (EPSI).” Psychological Assessment 25, no. 3: 859. 23815116 10.1037/a0032639

[eat24477-bib-0015] Forman‐Hoffman, V. 2004. “High Prevalence of Abnormal Eating and Weight Control Practices Among US High‐School Students.” Eating Behaviors 5, no. 4: 325–336. 15488447 10.1016/j.eatbeh.2004.04.003

[eat24477-bib-0016] Goldschmidt, A. B. , V. P. Aspen , M. M. Sinton , M. Tanofsky‐Kraff , and D. E. Wilfley . 2008. “Disordered Eating Attitudes and Behaviors in Overweight Youth.” Obesity 16, no. 2: 257–264. 18239631 10.1038/oby.2007.48

[eat24477-bib-0017] Gonsalves, D. , H. Hawk , and C. Goodenow . 2014. “Unhealthy Weight Control Behaviors and Related Risk Factors in Massachusetts Middle and High School Students.” Maternal and Child Health Journal 18, no. 8: 1803–1813. 10.1007/s10995-013-1424-5. 24357083

[eat24477-bib-0018] Hendricks, E. J. 2017. “Off‐Label Drugs for Weight Management.” Diabetes, Metabolic Syndrome and Obesity: Targets and Therapy 10: 223–234. 28652791 10.2147/DMSO.S95299 PMC5473499

[eat24477-bib-0019] Jo, T. 2019. Text Mining. Vol. 45. Springer International Publishing. 10.1007/978-3-319–91815-0.

[eat24477-bib-0020] Juarascio, A. , E. L. Lantz , A. F. Muratore , and M. R. Lowe . 2018. “Addressing Weight Suppression to Improve Treatment Outcome for Bulimia Nervosa.” Cognitive and Behavioral Practice 25, no. 3: 391–401. 10.1016/j.cbpra.2017.09.004. 30220839 PMC6132276

[eat24477-bib-0021] Kariuki, L. , C. Lambert , R. Purwestri , and H. K. Biesalski . 2016. “Trends and Consequences of Consumption of Food and Non‐Food Items (Pica) by Pregnant Women in Western Kenya.” NFS Journal 5: 1–4.

[eat24477-bib-0023] Loth, K. , R. MacLehose , M. Bucchianeri , S. Crow , and D. Neumark‐Stainer . 2014. “Personal and Socio‐Environmental Predictors of Dieting and Disordered Eating Behaviors From Adolescence to Young Adulthood: 10‐Year Longitudinal Findings.” Journal of Adolescent Health: Official Publication of the Society for Adolescent Medicine 55, no. 5: 705–712. 10.1016/j.jadohealth.2014.04.016. 24925491 PMC4380744

[eat24477-bib-0024] Monssen, D. , H. L. Davies , S. Kakar , et al. 2024. “The United Kingdom Eating Disorders Genetics Initiative.” International Journal of Eating Disorders 57, no. 5: 1145–1159. 37584261 10.1002/eat.24037

[eat24477-bib-0025] Morean, M. E. , and A. L'Insalata . 2017. “The Short Form Vaping Consequences Questionnaire: Psychometric Properties of a Measure of Vaping Expectancies for Use With Adult e‐Cigarette Users.” Nicotine & Tobacco Research 19, no. 2: 215–221. 27613904 10.1093/ntr/ntw205

[eat24477-bib-0026] Neumark‐Sztainer, D. , M. Wall , M. E. Eisenberg , M. Story , and P. J. Hannan . 2006. “Overweight Status and Weight Control Behaviors in Adolescents: Longitudinal and Secular Trends From 1999 to 2004.” Preventive Medicine 43, no. 1: 52–59. 16697035 10.1016/j.ypmed.2006.03.014

[eat24477-bib-0027] O'Connor, C. , N. McNamara , L. O'Hara , M. McNicholas , and F. McNicholas . 2021. “How Do People With Eating Disorders Experience the Stigma Associated With Their Condition? A Mixed‐Methods Systematic Review.” Journal of Mental Health 30, no. 4: 454–469. 31711324 10.1080/09638237.2019.1685081

[eat24477-bib-0028] Schaumberg, K. , A. Jangmo , L. M. Thornton , et al. 2019. “Patterns of Diagnostic Transition in Eating Disorders: A Longitudinal Population Study in Sweden.” Psychological Medicine 49, no. 5: 819–827. 29911514 10.1017/S0033291718001472 PMC6788452

[eat24477-bib-0029] Silge, J. , and D. Robinson . 2016. “Tidytext: Text Mining and Analysis Using Tidy Data Principles in R.” Journal of Open Source Software 1, no. 3: 37.

[eat24477-bib-0030] Solmi, F. , P. Sharpe , G. Suzanne Helen , J. Maddock , G. Lewis , and P. Patalay . 2021. “Changes in the Prevalence and Correlates of Weight‐Control Behaviors and Weight Perception in 49 Adolescents in the UK, 1986–2015.” JAMA Pediatrics 175, no. 3: 267–275. 10.1001/jamapediatrics.2020.4746. 33196811 PMC7670392

[eat24477-bib-0031] Song, Y. J. , J. H. Lee , and Y. C. Jung . 2015. “Chewing and Spitting out Food as a Compensatory Behavior in Patients With Eating Disorders.” Comprehensive Psychiatry 62: 147–151. 26343479 10.1016/j.comppsych.2015.07.010

[eat24477-bib-0032] Spindler, A. , and G. Milos . 2007. “Links Between Eating Disorder Symptom Severity and Psychiatric Comorbidity.” Eating Behaviors 8, no. 3: 364–373. 10.1016/j.eatbeh.2006.11.012. 17606234

[eat24477-bib-0033] Sysko, R. , D. R. Glasofer , T. Hildebrandt , et al. 2015. “The Eating Disorder Assessment for DSM‐5 (EDA‐5): Development and Validation of a Structured Interview for Feeding and Eating Disorders.” International Journal of Eating Disorders 48, no. 5: 452–463. 25639562 10.1002/eat.22388 PMC4721239

[eat24477-bib-0034] Thornton, L. M. , M. A. Munn‐Chernoff , J. H. Baker , et al. 2018. “The Anorexia Nervosa Genetics Initiative (ANGI): Overview and Methods.” Contemporary Clinical Trials 74: 61–69. 30287268 10.1016/j.cct.2018.09.015 PMC6338222

[eat24477-bib-0035] Udo, T. , and C. M. Grilo . 2018. “Prevalence and Correlates of DSM‐5–Defined Eating Disorders in a Nationally Representative Sample of U.S. Adults.” Biological Psychiatry 84, no. 5: 345–354. 10.1016/j.biopsych.2018.03.014. 29859631 PMC6097933

[eat24477-bib-0037] Westmoreland, P. , M. J. Krantz , and P. S. Mehler . 2016. “Medical Complications of Anorexia Nervosa and Bulimia.” American Journal of Medicine 129, no. 1: 30–37. 10.1016/j.amjmed.2015.06.031. 26169883

[eat24477-bib-0038] Yeatts, P. E. , S. B. Martin , T. A. Petrie , and C. Greenleaf . 2016. “Weight Control Behavior as an Indicator of Adolescent Psychological Well‐Being.” Journal of School Health 86, no. 8: 561–567. 10.1111/josh.12409. 27374345

