# Supplementary material for: Beyond the Diagnostic Checklist: A Large‐Scale Analysis of Under‐Recognized Weight Loss Behaviors in Individuals With Eating Disorders
Source: Int J Eat Disord. 2025 Jun 17;58(9):1806–16. doi: 10.1002/eat.24477 (PMC12423573; doi:10.1002/eat.24477)
Supplement: Supplementary file 1 — Data S1.Supporting Information. [file EAT-58-1806-s001.docx]

## **Beyond the Diagnostic Checklist: A large-scale analysis of under-recognised weight loss behaviours in individuals with eating disorders**

## Saakshi Kakar^1,2^, Una Foye^1,3^ , Helena L. Davies^1,2, 4,5^ , Elisavet Palaiologou^1^, Chelsea M. Malouf^1,2^ , Laura Meldrum^1,2^, Iona Smith^1,2^, Gursharan Kalsi^1,2^, Karina L. Allen^6,7^, Gerome Breen^1,2^ , Moritz Herle*^1^ & Christopher Hübel*^1,2,8,9^

## Supplementary Methods

**Online data collection**

Participants were recruited via social media campaigns and through participating UK National Health Service (NHS) sites. Individuals enrolled in the study by registering and providing informed consent through the study websites (gladstudy.org.uk; edgiuk.org). As part of the registration process, participants were required to authenticate their accounts by entering a verification code sent to their email. Upon successful registration, participants completed the study questionnaire hosted on the Qualtrics platform. To ensure data integrity, bot detection features were enabled within Qualtrics to identify and filter out suspicious or automated responses.
